# Effect of Different Durations of Eye-Covering Pretreatment on Emergence Delirium after Ophthalmic Surgery in Preschool-Aged Children: A Randomized Controlled Study

**DOI:** 10.1155/2022/3656148

**Published:** 2022-09-16

**Authors:** Pei-Fang Dong, Dan-Ni Qiao, Hui-Lian Chen, Shui-Hua Lu, Shao-Hong Qu, Yun-Tao Wu, Di Zhao, Ting Wan

**Affiliations:** ^1^Nursing Department of Eye Center, The Second Affiliated Hospital Zhejiang University School of Medicine, Hangzhou 310009, Zhejiang, China; ^2^Eye Center, The Second Affiliated Hospital Zhejiang University School of Medicine, Hangzhou 310009, Zhejiang, China

## Abstract

**Background:**

Preoperative eye-covering training for 3 hours has been reported to effectively reduce the incidence of emergence delirium (ED) in preschool children. However, most children can only maintain the eye being covered for less than 60 min, and shortening eye-covering duration can also achieve similar clinical effects as long duration of eye-covering. This study was designed to compare the effects of 30-min and 60-min eye-covering pretreatment based on cartoon education only on preoperative anxiety, postoperative ED, and pain score after ophthalmic surgery with general anesthesia in preschool-aged children.

**Methods:**

Preschool-aged children (3–7 years) who were diagnosed with cataract, blepharoptosis, trichiasis, strabismus, eyelid tumor, and underwent ophthalmic surgery with general anesthesia from August 2021 to January 2022 were recruited. A total of 228 patients were randomly assigned at a 1 : 1:1 ratio to receive 30-min eye covering (30-min group), 60-min eye covering (60-min group) pretreatment, or programmed education only (C group). The preoperative anxiety, postoperative emergence delirium, and pain were compared between the groups.

**Results:**

The preoperative anxiety score, postoperative ED score, and incidence of ED in the 30-min group (*n* = 76) and 60-min group (*n* = 72) were significantly lower than those in the C group (*n* = 76), demonstrating a significant between-group difference (*P* < 0.001). However, the 30-min group and 60-min group had no significant difference in the abovementioned outcome measures (*P* > 0.05). Moreover, no significant difference was found in postoperative pain scores among the three groups (*H* = 0.274, *P*=0.872).

**Conclusion:**

Both 30-min and 60-min eye-covering pretreatments significantly reduce preoperative anxiety and postoperative ED after ophthalmic surgery with general anesthesia in preschool-aged children. The effects of the two groups show no intergroup difference, but the 30-min eye-covering pretreatment may be more convenient for practicing. *Trial Registration*. This study was registered with the No. NCT04973150.

## 1. Introduction

The incidence of emergence delirium (ED) after general anesthesia varies from 25% to 80% in preschool-aged children [1–3] and is particularly high in children receiving ophthalmic surgery. The reason is that eye dressings used after ophthalmic surgery result in darkness and visual disturbance, triggering patients' fear, and thus, a high level of psychological stress [4–6]. ED causes agitation, thereby increasing the difficulty of clinical care and inducing potential safety hazards, such as damage to the surgical site and venous access. In short, ED is a clinical problem that needs to be solved urgently.

Kain et al. [7] pointed out that ED after general anesthesia in children was related to preoperative anxiety; the risk rate of ED increases 10% for every 10-point increase in the preoperative anxiety score. Preschool children are still in the immature stage of cognitive development. Because of that, common preoperative oral education is not enough for them to understand the purpose and the significance of the surgery, making it hard to efficiently decrease their preoperative anxiety levels. Dai et al. found that scenario simulation enhanced the children's personal control over the procedure, contributing to reducing preoperative fear and anxiety [8]. Lin et al. [4] revealed that preoperative eye-covering training for 3 h in preschool children effectively reduced the incidence of ED after cataract surgery with general anesthesia. Eye-covering pretreatment is an effective nonpharmaceutical intervention method to simulate the postoperative visual state using an eye mask before surgery, and importantly, it is easy to implement. However, preschool children are active, have poor self-control, and always have low compliance with continuous eye covering for more than 3 h, which is a great challenge for clinical applications.

According to prospective cohort observations by Dong et al. [9], eye-covering pretreatment with a low duration (30–89 min) and a medium duration (90–180 min) could also achieve similar clinical effects compared to the high-duration group (≥3 h), with no significant differences among the three groups. Moreover, most of the children could only keep the eye covered for less than 60 min. Considering the cognitive and concentration characteristics of preschool-aged children, we only compared the effects of eye-covering pretreatment lasting 30 min and 60 min on the incidence of ED after ophthalmic surgery with general anesthesia in preschool-aged children. This study aimed to determine the more suitable eye-covering duration that would be easy for children to accept and convenient for the nursing staff to operate.

## 2. Methods

### 2.1. Study Design

This prospective, randomized controlled study was conducted at a university hospital eye center in Zhejiang, China, from August 2021 to January 2022. A parallel trial design was used, and the children received 30-min eye-covering pretreatment (30-min group), 60-min eye-covering pretreatment (60-min group), and no eye-covering pretreatment (C group), respectively. This study was guided by the CONSORT checklist [10] (Supplementary Table 1). The study has been prospectively registered at https://register.clinicaltrials.gov.

### 2.2. Participant Eligibility Criteria

Patients were enrolled in the trial if they met following inclusion criteria: (a) preschool children aged 3–7 years; ((b) underwent scheduled ophthalmic surgery with general anesthesia; (c) first experience of surgery; (d) the diagnosis and surgery type: cataract (phacoemulsification and intraocular lens implantation), blepharoptosis (frontalis suspension), trichiasis (Hotz procedure), strabismus (strabismus surgery), and eyelid tumor (eyelid tumor excision). Patients were excluded from the trial if they reported the following: (a) refusal in receiving eye-covering pretreatment; (b) attention deficit disorder; (c) binocular vision impairment; (d) eyeball removal and/or orbital implantation; (e) history of taking psychotropic drugs; (f) inability to communicate normally; (g) mild cold and cough; (h) and severe heart, lung, brain, kidney, and other organ diseases. Some eligible patients were also excluded from the final analysis if the following situations occurred: (a) failure to complete the designated eye-covering time; (b) duration of anesthesia of more than 60 min; (c) sevoflurane inhalation anesthesia; (d) complications such as anesthetic allergy occurring during the operation; (e) incomplete data.

### 2.3. Sample Size

The main observation outcome was the incidence of ED. According to the pre-experimental results of eye-covering pretreatment and related literature [1], the incidence of ED was 40%, 39%, and 70% in the 30-min group, 60-min group, and C group, respectively. According to a 1 : 1:1 parallel control, the sample size was calculated based on a power calculation (*α* = 0.05, *β* = 0.90) using PASS 15.0 software. According to the calculation, the sample size for each group was 68 cases, and to account for a 10% dropout in the follow-up, 76 patients were recruited for each group.

### 2.4. Randomization and Data Collection

Two hundred and twenty-four preschool children were enrolled in the study. A single researcher (called D) gave information about the study to the participants in the eye center unit, and the informed consent was obtained from children's parents. The allocation concealment was guaranteed by a computer-generated number table by researcher D. Children and their parents were all blinded to know which group they were enrolled. Researcher D informed researcher C which groups the children were allocated by an opaque envelope. Then, researcher C was responsible for having children practice eye-covering pretreatment one day before the operation in the eye unit. The evaluation of preoperative anxiety (m-YPAS, the Modified Yale Preoperative Anxiety Scale) was completed by an anesthesiologist (called W), while the assessment of postoperative ED (NU-DESC, the Nursing Delirium Screening Scale) and pain (FPS-R, the Wong–Baker Facial Pain Rating Scale) were administered by researcher Q. They were all blinded to the group assignment.

### 2.5. Primary Outcomes

The primary outcomes were the score and incidence of ED. The Nursing Delirium Screening Scale [11] was used to score ED in five areas: disorientation, inappropriate behavior, inappropriate communication, delusions/hallucinations, and psychomotor retardation. Each feature was graded based on severity: 0 = absent, 1 = mild, and 2 = moderately severe. The total score was 0–10, and delirium was diagnosed when the total score was ≥2. The higher the score, the higher the degree of ED in children. The incidence of ED was calculated as follows: number of children with a score ≥2 points/total number of children × 100%. The evaluation was completed by researcher Q within 2 h after surgery.

### 2.6. Secondary Outcome

The secondary outcomes were the preoperative anxiety score and the postoperative pain score. The preoperative anxiety levels were evaluated by the modified Yale Preoperative Anxiety Scale [12] which was typically used in children aged 2–12 years. The questionnaire includes five domains and 22 items, including activity (four items), vocalization (six items), emotional expressivity (four items), state of apparent arousal (four items), and use of parents (four items). Each item was rated as 1–4 points except the vocalization domain (1–6 points). The scale was converted to a percentile system, with a minimum value of 0 points and a maximum value of 100 points. A higher score indicated a higher anxiety level of a child. The evaluation was performed by anesthesiologist W when the child entered the operating room for about 2 min.

For the pain score, the Wong–Baker Facial Pain Rating Scale [13] was adopted, a simple and intuitive assessment method mainly applicable to children aged three years and above. In this scale, different facial expressions are used to represent different degrees of pain. Each child chose a face that best represented his or her own pain level and pain severity was rated by the score corresponding to the face. Researcher Q determined the frequency of assessment based on the child's pain and recorded the highest score within 6 h after the operation.

General information, including children's age, gender ratio, diagnosis, duration of surgery, surgical eye type, and education level of parent or guardian, were collected when patients were enrolled.

### 2.7. Intervention

Eye-covering pretreatment was performed by researcher C (ophthalmology clinical nurse specialist) in children's activity room which was completed separately. Using a random number table, the enrolled children were randomized into the C group (programmed education only), 30-min group (30-min eye-covering), and 60-min group (60-min eye-covering), respectively.

A preoperative education via a cartoon animation video (3.5 min) was carried out for all the children on a tablet computer one day before the operation. We chose the children's favorite cartoon characters, “Rabbit” and “Doctor Elephant”, and used “magic” as a keyword, to introduce the children's identity (magic wristband), surgical mark (magic sign), eye medicine (magic medicine), anesthesia surgery process (magic sleep), postoperative gauze covering (magic goggles), and pain education (magic mask). Prevention of accidental falls and burns, and other perioperative educational content were also included. After preoperative education, the children were asked to make sure that they understood the content of the video.

After the video was played, the children in the 30-min group and 60-min group were trained to cover one or both eyes with gauze depending on the type of surgery. Children with unilateral eye covering could move around with their parents or nursing staff; children with bilateral eye-covering rested in bed, listened to music or stories, or performed appropriate activities with the support of their parents. To improve the completion rate of eye-covering training, the children were encouraged in the form of alternate rewards of candies and toys. Material rewards and verbal praise were given once every 30 min. Safety protection during the pretreatment process included providing a safe training environment without obstacles or dangerous objects and being accompanied by the nursing staff or their parents during the activity. Additionally, hospital beds had bed rails on both sides for protecting children undergoing binocular eye-covering training. The training ended immediately once the child panics or cries with ineffective comforting.

Prior surgery, the patients abstained from solid food for 8 h, milk for 6 h, and clear liquids for 2 h and received no preanesthetic medication. After arriving in the operating room, the patients were regularly monitored for the following: electrocardiograph, heart rate (HR), noninvasive blood pressure (NIBP), peripheral capillary oxygen saturation (SpO2), Bispectral Index (BIS), and end-tidal carbon dioxide. Three minutes after preoxygenation, all patients were anesthetized intravenously with 2 *μ*g/kg of fentanyl citrate and 0.5–2 mg/kg of propofol at doses determined by the anesthesiologist, and a laryngeal mask airway was inserted after 0.15 mg/kg of cisatracurium. Adjustable mechanical ventilation was regulated to keep the end-tidal carbon dioxide level at 30–40 mmHg. Anesthesia was maintained by continuous intravenous infusion of remifentanil (40 mcg/ml) and 2% propofol (20 mg/ml) using a micropump to ensure BIS values of 45–55.

The anesthesia was stopped near the end of the operation, and the laryngeal mask airway was removed when sufficient spontaneous breathing was restored and the BIS was above 80. The patient was then transferred to the postanesthesia care unit (PACU) for further observation. The patients' HR, NIBP, and SpO2 levels were continuously monitored by PACU nurses and they used masks with an oxygen flow of 2 L/min until the patients regained consciousness. All the children were discharged from the PACU when they were fully awake with stable vital signs, an oxygen saturation of more than 95%, and no vomiting or agitation.

### 2.8. Statistical Analysis

The measurement data were tested for normal distribution. Data conforming to a normal distribution and homogeneity of variance were described using the mean and standard deviations, and one-way analysis of variance was used to examine the differences between each group. Nonnormally distributed data were represented by median and quartile, and comparison between the groups was achieved using the Kruskal–Wallis rank-sum test. Count data were described using frequency and percentage, and the comparison between the groups was performed by the *χ*2 test. SPSS 23.0 software package was used to conduct the statistical analysis. A *P* value of <0.05 was considered statistically significant.

### 2.9. Ethical Considerations

This prospective research adhered to the Helsinki Declaration and was approved by the hospital's ethics committee ((2020) No. 527). The parents (or guardians) of all the participants signed informed consent forms.

## 3. Results

**Figure 1:**
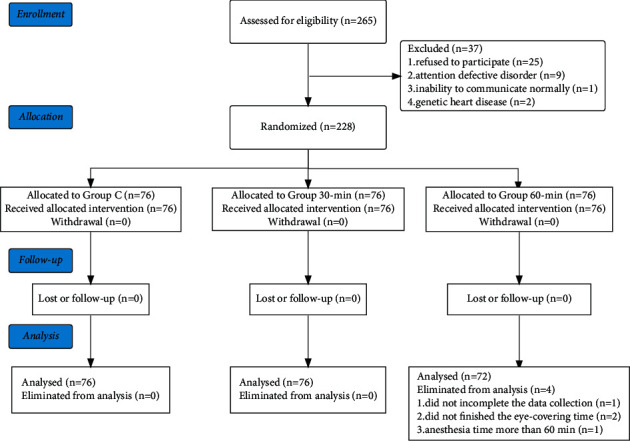
Flowchart of the enrollment data.

Figure 1 represents the flowchart of the study. A total of 265 cases were assessed for eligibility, of which 37 cases were excluded for the following reasons: refusal to participate (*n* = 25), attention deficit disorder (*n* = 9), inability to communicate normally (*n* = 1), and genetic heart disease (*n* = 2). Overall, 228 cases were recruited into the study, however, four of whom were eliminated from the 60-min group for the following reasons: two cases did not finish 60-min eye-covering, one case had an anesthesia time of more than 60 min, and one case gave incomplete data. Thus, a total of 224 cases were included in the final analysis (C group, *n* = 76; 30-min group, *n* = 76; 60-min group, *n* = 72). Patients in the 30-min and 60-min groups showed good compliance during training (30-min group: 100%; 60-min group: 97.3%), and no injury occurred during the training.

There were no statistically significant differences in age, gender ratio, types of diagnosis, and duration of surgery among the three groups (*P* > 0.05; Table 1). The education level of the parents or guardians and the type of surgical eye (monocular/binocular) were similar in three groups (*P* > 0.05; Table 1). The preoperative anxiety score in the 30-min group and the 60-min group were lower than those in the C group (40.90 (31.81,45.45), 40.90 (31.81,50.00) vs 50.00 (45.45,63.63)), demonstrating a significant between-group difference (*H* = 29.638, *P* < 0.001, Table 2). Interestingly, the postoperative incidence of ED in the 30-min group and the 60-min group were markedly decreased as compared with the C group (26.3%, 34.7% vs 77.6%, *χ*^ 2^ = 46.078, *P* < 0.001). Meanwhile, a significant difference was also found in the postoperative ED score among the three groups (1.0 (0.00,2.00), 1.0 (0.00,2.00) vs 2.5 (2.00,4.00), *H* = 44.785, *P* < 0.001), but not in the postoperative pain score (*H* = 0.274, *P* > 0.05), as shown in Table 2. Furthermore, the 30-min group and 60-min group had no marked difference in preoperative anxiety score, postoperative ED score, and incidence of ED (all *P* > 0.05), as shown in Table 3.

## 4. Discussion

This study investigated the effects of different durations of eye-covering pretreatment on ED after ophthalmic surgery with general anesthesia in preschool-aged children. We found that the preoperative anxiety score, postoperative ED score, and incidence of delirium in the 30-min group and 60-min group were lower than those of the C group, and that the differences were statistically significant. However, no significant difference was found between the 30-min group and 60 min group in these outcome measures (all *P* > 0.05). No significant difference was identified in the postoperative pain score among the three groups.

The incidence of ED after general anesthesia in preschool children who underwent ophthalmic surgery is the result of a multifactor synergistic effect, including preoperative anxiety, postoperative pain, anesthesia medication, duration of anesthesia, and visual disturbance caused by eye covering after surgery [2, 4, 7, 14–17]. Among these factors, anesthesia duration and postoperative pain are considered the two most important. To evaluate the effects of different eye-covering durations on the incidence of ED, we excluded cases with an anesthesia duration of more than 1 h to eliminate the difference in anesthesia duration. Our results also demonstrated no significant difference in postoperative pain scores among the three groups. According to comparison results, both 30-min eye-covering and 60-min eye-covering could significantly decrease preoperative anxiety and the incidence of ED after ophthalmic surgery with general anesthesia. This may be because the cartoon educational video combined with eye-covering pretreatment enables the children to experience and adapt to the state of postoperative eye-covering. This strategy reduces the psychological stress triggered by the sudden and long-lasting darkness experienced after waking up from general anesthesia. It also decreases the anticipatory anxiety and fear caused by the unknown and the state of uncertainty before surgery. The obtained results by this trial are consistent with previous reports that an eye-covering pretreatment intervention could effectively reduce the incidence of ED after general anesthesia in children by decreasing preoperative anxiety and visual disturbance [1, 2, 4, 18–21].

As a nonpharmaceutical intervention, preoperative eye-covering training is simple, safe, and effective. However, the problem occurring during the training is that children's compliance with continuous eye-covering for more than 3 h is low. We previously conducted a prospective cohort study to compare the effects of eye-covering pretreatment on the incidence of ED in short (30–89 min), medium (90–179 min), and long durations (≥3 h). Even with games as the entry point and candy or toys to encourage children, most of them could only keep their eye (*s*) covered for less than 60 min. The encouraging result is that in the three cohorts of the low-, medium-, and long-duration groups, the incidence of ED was all effectively reduced after general anesthesia in preschool children who underwent ophthalmic surgery, without a statistically significant intergroup difference. Thus, in this study, we further compared the effects of 30-min and 60-min eye-covering pretreatments for reducing ED after general anesthesia and found that 100% of the patients in the 30-min group and 97% of the patients in the 60-min group completed their eye-covering training. Furthermore, the postoperative ED score and incidence of ED in the two groups were similar. Preschool children are only able to maintain their concentration for 15 to 20 min during quiet activities (e.g., reading books) and for 20 to 30 min during free and open activities [22–24]. The eye-covering activity helps children form a vivid and comprehensive memory of visual disturbance, which is consistent with the cognitive level and memory characteristics of preschool children [25, 26]. The repetitive inhibition effect is prone to produce if the continuous eye-covering training method used in this study is under the intensive learning method. Additionally, brain imaging studies have found that, compared to the first presentation of the stimulus, the activation of specific brain regions decreases when the stimulus is presented repeatedly [27]. Thus, it seems that a longer pretreatment time for eye covering will not be superior to a shorter pretreatment time in reducing the incidence of ED after general anesthesia. Collectively, 30-min eye-covering training is recommended, which can improve children's compliance and reduce the operator's time cost without affecting the clinical effect.

Our study has some limitations. First, the outcome measures are not comprehensive enough, which only include scale scores but no physiological indexes. Second, this is a single-center study, so multicenter and large sample studies are still required. In the future, we plan to compare the effects of decentralized and concentrated eye-covering training on ED incidence to determine a more scientific, reasonable, and practical training method for clinical use.

## 5. Conclusion

Eye-covering pretreatment is a safe, simple, effective, and low-cost nonpharmaceutical intervention with great clinical value in reducing the incidence of ED after general anesthesia in preschool children who have undergone ophthalmic surgery. Both 30-min and 60-min eye-covering pretreatments can significantly reduce preoperative anxiety and postoperative ED after ophthalmic surgery with general anesthesia in preschool-aged children. The two groups had no significant difference in the abovementioned outcome measures, but the 30-min eye-covering pretreatment is more convenient for patients and medical staff.

## Figures and Tables

**Table 1:** Baseline characteristics of study subjects.

Variables	*C* group (*n* = 76)	30-min group (*n* = 76)	60-min group (*n* = 72)	F/*χ*^ 2^	*p*
Age (yr)	4.55 ± 1.44	4.85 ± 1.46	4.68 ± 1.46	0.828^^^	0.438
Gender, M/F	40 (52.6)/36 (47.4)	38 (50.0)/38 (50.0)	45 (62.5)/27 (37.5)	2.575^#^	0.276

Diagnosis				7.480^#^	0.486
Cataract	12 (15.8)	6 (7.9)	9 (12.5)		
Blepharoptosis	20 (26.4)	22 (28.9)	27 (37.5)		
Trichiasis	21 (27.6)	21 (27.7)	11 (15.3)		
Strabismus	21 (27.6)	25 (32.9)	22 (30.5)		
Eye lid tumor	2 (2.6)	2 (2.6)	3 (4.2)		

Duration of surgery (min)	39.21 ± 6.87	37.39 ± 8.97	38.08 ± 8.41	0.964^^^	0.383
Parent/Guardian education level				2.583^#^	0.859
Bachelor or above	47 (61.8)	49 (64.4)	39 (54.2)		
High school	12 (15.8)	10 (13.2)	14 (19.5)		
Junior high school	15 (19.8)	15 (19.8)	18 (25.0)		
Primary school and below	2 (2.6)	2 (2.6)	1 (1.3)		

Operative eye (%)				0.092^#^	0.955
Monocular	34 (44.7)	35 (46.1)	34 (47.2)		
Binocular	42 (55.3)	41 (53.9)	38 (52.8)		

Data are *n* (%) unless otherwise stated. Percentages might not add to 100% due to rounding;  ^: One-way ANOVA test used to calculate F value; #: Chi-square test used to calculate *χ*^ 2^ value.

**Table 2:** Differences in overall study outcomes between three groups.

Variables	C group (*n* = 76)	30-min group (*n* = 76)	60-min group (*n* = 72)	*χ* 2/H	*p*
Preoperative anxiety score (IQR)	50.00 (45.45, 63.63)	40.90 (31.81, 45.45)	40.90 (31.81, 50.00)	29.638^&^	<0.001
Postoperative pain score (IQR)	0.00 (0.00, 0.00)	0.00 (0.00, 0.00)	0.00 (0.00, 0.00)	0.274^&^	0.872
Postoperative ED score (IQR)	2.5 (2.00, 4.00)	1.0 (0.00, 2.00)	1.0 (0.00, 2.00)	44.785^&^	<0.001
Incidence of ED (%)	59 (77.6)	20 (26.3)	25 (34.7)	46.078^#^	<0.001

Data are *n* (%) unless otherwise stated. Percentages might not add to 100% due to rounding; IQR = interquartile range; #: Chi-square test used to calculate *χ*2 value; &: Kruskal–-Wallis test used to calculate *H* value.

**Table 3:** *p* values of the inter-group comparison among the three groups.

Comparison	Preoperative anxiety score	Postoperative pain score	Incidence of ED (%)	Postoperative ED score
C Group vs 30-min group	<0.001	0.395	<0.001	<0.001
C Group vs 60-min group	<0.001	0.608	<0.001	<0.001
30-min group vs 60-min group	0.937	0.744	0.255	0.516

One-way ANOVA least significant difference test used to calculate *p* value.

## Data Availability

The data that support the findings of this study are available from the corresponding author upon reasonable request.

## Supplementary Materials

Supplementary Materials Supplementary Table 1: CONSORT 2010 checklist of information to include when reporting a randomized trial.
